# An analytical approach for unsupervised learning rate estimation using rectified linear units

**DOI:** 10.3389/fnins.2024.1362510

**Published:** 2024-04-08

**Authors:** Chaoxiang Chen, Vladimir Golovko, Aliaksandr Kroshchanka, Egor Mikhno, Marta Chodyka, Piotr Lichograj

**Affiliations:** ^1^School of Information Science and Technology, Zhejiang Shuren University, Hangzhou, China; ^2^International Science and Technology Cooperation Base of Zhejiang Province: Remote Sensing Image Processing and Application, Hangzhou, China; ^3^Institute of Traditional Chinese Medicine Artificial Intelligence Zhejiang Shuren University, Hangzhou, China; ^4^Department of Computer Science, John Paul II University in Biala Podlaska, Biala Podlaska, Poland; ^5^Intelligent Information Technologies Department, Brest State Technical University, Brest, Belarus

**Keywords:** adaptive training step, RBM, deep learning, unsupervised learning, ReLU, activation function, Adam

## Abstract

Unsupervised learning based on restricted Boltzmann machine or autoencoders has become an important research domain in the area of neural networks. In this paper mathematical expressions to adaptive learning step calculation for RBM with ReLU transfer function are proposed. As a result, we can automatically estimate the step size that minimizes the loss function of the neural network and correspondingly update the learning step in every iteration. We give a theoretical justification for the proposed adaptive learning rate approach, which is based on the steepest descent method. The proposed technique for adaptive learning rate estimation is compared with the existing constant step and Adam methods in terms of generalization ability and loss function. We demonstrate that the proposed approach provides better performance.

## 1 Introduction

During recent years many papers have been devoted to the study of restricted Boltzmann machines (RBM) and more generally to that of deep learning, because it is a breakthrough approach in the field of artificial intelligence (Hinton, 2002, 2010; Hinton et al., 2006; Hinton and Salakhutdinov, 2006; Nair and Hinton, 2010; Krizhevsky et al., 2012; LeCun et al., 2015). Deep learning has been developing very quickly in the last decade. As a result, various successful applications of deep learning have been proposed in speech recognition, computer vision, natural language processing, data visualization, etc. (Bengio et al., 2007, 2013, 2021; Bengio, 2009; Larochelle et al., 2009; Erhan et al., 2010; Golovko et al., 2010; Glorot et al., 2011; Mikolov et al., 2011; Hinton et al., 2012; Madani et al., 2018; Lamb et al., 2022; Verma et al., 2022; Aguilera et al., 2023; Menezes et al., 2023; Chen et al., 2024).

One of the major and important problems in this domain is the selection of suitable hyperparameters values to achieve significant performance of a neural network. Among these parameters, the learning rate is of great importance because it has a significant impact on the training efficiency of the neural network (Golovko, 2003; Cho et al., 2011; Duchi et al., 2011; Krizhevsky and Hinton, 2012; Zeiler, 2012; Schaul et al., 2013; Kingma and Ba, 2014; Ruder, 2016; Pouyanfar and Chen, 2017; Smith, 2017; Baydin et al., 2018; Takase et al., 2018; Arpit and Bengio, 2019; Vaswani et al., 2019; Pesme et al., 2020; Carvalho et al., 2021; Nakamura et al., 2021; Chen et al., 2022; Defazio et al., 2023; Golovko et al., 2023; Wang et al., 2023). The choice of an appropriate learning rate controls how well the neural network adapts to the problem being solved and achieves a suitable minimum of the loss function. So, for instance, for many applications the learning rate has to be manually and carefully chosen, because depending on this parameter the learning process can be divergent or convergent. Therefore, to avoid these problems, learning step should be defined and modified automatically during neural network learning.

The neural networks community has been concerned with this problem for many years, and currently there are only partial solutions to selecting an appropriate learning rate. This situation gives rise to the question of how we can obtain analytical expressions for learning rate calculation. This question is addressed in the present paper. As a result, an analytical approach to estimate the value of the learning step has been proposed, based on the steepest descent approach. The proposed approach capables to automatically defining and adjusting the learning rate during the training of a neural network.

In our previous work (Golovko et al., 2023), we proposed an approach to estimate the learning rate of a single-layer perceptron with a rectified linear unit activation function (ReLU). The present article focuses on an adaptive learning step (ATS) for RBM with a ReLU. It is the simplest activation function, which is a piecewise linear function consisting of two straight lines. ReLU is not a saturated activation function with unlimited output, unlike other activation functions. It has been noted in existing literature that using a ReLU network generally improves performance (Vaswani et al., 2019; Wang et al., 2023). As stated in the article (Nair and Hinton, 2010) rectified linear units can improve RBM. As well is known a RBM can be applied for deep neural networks learning (LeCun et al., 2015). The conventional approach to RBM learning usually uses constant or empirically varying learning step (Cho et al., 2011). Currently, there are no analytical expressions to estimate the learning rate, which can be automatically defining and adjusting the learning rate during the training of a RBM network. As a rule, there are only empirical and heuristic approaches to set learning rate.

Therefore, in this paper we investigate the calculation of adaptive learning rate for a RBM, which is based on the steepest descent technique (Golovko et al., 2000, 2023; Golovko, 2003). This approach is based on minimizing the loss function to calculate the adaptive learning step. Since derivation an accurate analytical expression for estimating the learning rate using steepest descent approach is a very difficult task, most scientists use the steepest descent method together with the line search approach. However, as we will show in this article, it is possible to derive exact expressions for the RBM learning rate using the ReLU activation function. The adaptive learning rate approach permits to compute the learning step at each time. An advantage of the proposed approach is that we can automatically estimate a specific learning rate value for each batch or each example from the training data set.

Further, we perform stacking ReLU RBM into a deep neural network. As a result, we can train deep neural networks using unsupervised and SGD techniques.

The major contribution of this paper is novel mathematical expressions for adaptive learning rate calculation, if we use RBM with ReLU transfer function. The proposed approach is based on steepest descent technique and allows to estimate the ATS at each iteration of the learning algorithm. We have shown, using a set of experiments, that the proposed adaptive learning rate can improve performance with respect to learning quality and generalization ability.

In the present study we proceed as follows. Section 2 introduces the related work in this area. In Section 3 we consider different representations of RBM. Section 4 deals with learning rules for RBM with ReLU. In section 5 we propose the adaptive learning step calculation for RBM. Section 6 demonstrates the results of experiments, and finally we give our conclusion.

## 2 Related work

In the following, a brief overview of related works in this area is presented. It is well known that there are the two principal techniques for learning of deep neural networks (DNN): learning with pretraining using a greedy layer wise approach and stochastic gradient descent approach (SGD), including its various modifications. If we do not use pretraining of DNN, then it is necessary to use a rectified linear unit (ReLU) transfer function, because of the vanishing gradient problem (LeCun et al., 2015).

RBM can be used as building blocks for deep neural networks, where every layer of neural network is trained as RBM in an unsupervised manner (Hinton, 2002, 2010; Hinton et al., 2006; Hinton and Salakhutdinov, 2006; Nair and Hinton, 2010). By stacking RBMs in this way, one can obtain a suitable initialization of a deep neural network for further training using a backpropagation algorithm.

For smaller data sets, unsupervised pretraining helps to prevent overfitting (LeCun et al., 2015). As stated in paper (LeCun et al., 2015): “Although at present the supervised training with ReLU is used mainly for deep neural networks learning, we expect unsupervised learning to become far more important in the longer term. Human and animal learning is largely unsupervised: we discover the structure of the world by observing it, not by being told the name of every object.” Consequently, unsupervised learning is of great importance. Therefore, we consider in this work the different representations of RBM and study estimation of an adaptive learning rate.

Currently the most methods for learning rate estimation are oriented to the SGD approach (Duchi et al., 2011; Zeiler, 2012; Schaul et al., 2013; Kingma and Ba, 2014; Ruder, 2016; Pouyanfar and Chen, 2017; Smith, 2017; Baydin et al., 2018; Takase et al., 2018; Vaswani et al., 2019; Nakamura et al., 2021; Chen et al., 2022; Defazio et al., 2023; Wang et al., 2023). If the SGD approach is used, then, as a rule, an initial learning rate is selected manually, and further during the learning, the training rate is decreased over time, using different rules. We have not found any works as concerns analytical expressions for the learning rate estimation. There are various approaches to learning rate estimation using different versions of SGD. Let us consider these approaches shortly.

Existing works related to learning rate selection are based mostly on learning rate schedule or line search approach. So, for instance the estimation of adaptive learning rate using line search approach is proposed in Vaswani et al. (2019) and Wang et al. (2023). As mentioned earlier, as a rule, the line search approach is used in conjunction with the steepest descent technique. However, such an approach is computationally expensive and time consuming. Furthermore, as will be shown in this paper, it is possible to obtain for RBM with ReLU precise expressions for the learning rate instead of using line search. Learning rate scheduling is a very popular approach and is used in various gradient descent optimization algorithms, namely, Adagrad, Adadelta, RMSprop, and Adam. The primary shortcoming associated with learning rate schedules is their dependence on predefined initial learning rate.

So, for instance, the Adagrad method (Duchi et al., 2011) divides the learning rate at each step by the norm of all previous gradients. The other approaches, such as Adadelta and Adam are based on Adagrad and as a result the learning rate decreases during training (Kingma and Ba, 2014; Ruder, 2016). In Pesme et al. (2020), the optimization process of SGD is divided into two stages: transient stage and stationary stage. It should be noted that the learning step is reduced during the stationary phase. In Smith (2017), scheduling learning rate is performed for each iteration. In Baydin et al. (2018), the hypergradient descent approach is proposed in order to find appropriate learning step. In Nakamura et al. (2021), ATS technique is proposed, which is based on a combination of reducing and increasing the learning rate.

As regards analytical learning rate at the pretraining stage, we have not found any works as concerns the learning rate estimation. Substantially, all known approaches are based again not on analytical expressions for calculating the learning rate, but on empirical approaches and the policy of changing the learning step. So, for instance, in Cho et al. (2011) for RBM is proposed an approach to automatically adjust the learning rate by maximizing a local likelihood estimate. However, as a result, the learning rate is chosen based on the previous learning rate and a small constant, that leads again in manual selection of the initial parameters.

In this paper we propose to use steepest descent approach to derive learning rate. Such learning rate can only be obtained for linear and ReLU activation functions. When using the sigmoid activation function, we can only receive approximate expressions for the learning rate using the Taylor series expansion (Golovko et al., 2000; Golovko, 2003). Since this is a very complicated problem, as mentioned before, most of the scientists use the steepest descent method together with the line search approach.

Our previous work (Golovko et al., 2023) reported an adaptive learning rate for a single-layer perceptron with a ReLU activation function. Let us consider the simplest neural network, namely single layer perceptron (SLP). In the case of a single-layer perceptron with ReLU activation function, the expressions for calculating the adaptive learning step was obtained for the first time in the work (Golovko et al., 2023) based on the proof of the following theorems:

**Theorem 1:** For a single-layer perceptron with a ReLU activation function in the case of online learning, the value of the adaptive learning step is calculated based on the following expression Eq. (1):


(1) $$\alpha \left(t\right)=\frac{\sum_{j=1}^{m}r_{j}\left(t+1\right)b_{j}\left(r_{j}\left(t+1\right)S_{j}\left(t\right)-e_{j}\right)}{\sum_{j=1}^{m}r_{j}^{2}\left(t+1\right)b_{j}^{2}}$$

$$b_{j}=r_{j}\left(t\right)\left(y_{j}-e_{j}\right)\left(1+\overset{n}{\underset{i=1}{\sum }}x_{i}^{2}\left(t\right)\right)\text{,}$$

where $r_{j}\left(t+1\right)=\{\begin{array}{c} r_{1},e_{j}\left(t\right)\geq 0;\\ r_{2},e_{j}\left(t\right)<0. \end{array}$

Here $r_{1}$ and $r_{2}$ denotes corresponding slopes of the ReLU function; $e_{j}\left(t\right)$ is desired output for j-th unit; n and m denotes the number of input and output unit, $S_{j}\left(t\right)$ , $y_{j}\left(t\right)$ are weighted sum and output of the j-th unit.

It should be noted, that $r_{1}\neq r_{2}$ and 0 < r_2_ < 1.

**Theorem 2:** For a single-layer perceptron with a ReLU activation function in the case of batch learning, the value of the adaptive learning step is calculated based on the following expression Eq. (2):


(2) $$\alpha \left(t\right)=\frac{\sum_{k=1}^{L_{1}}\sum_{j=1}^{m}\left(r_{j}^{k}\left(t+1\right)S_{j}^{k}\left(t\right)-e_{j}^{k}\right)\left(r_{j}^{k}\left(t+1\right)b_{j}^{k}\right)}{\sum_{k=1}^{L_{1}}\sum_{j=1}^{m}{\left(r_{j}^{k}\left(t+1\right)b_{j}^{k}\right)}^{2}}$$

$$b_{j}^{k}=\overset{L_{1}}{\underset{p=1}{\sum }}r_{j}^{p}\left(t\right)\left(y_{j}^{p}-e_{j}^{p}\right)\left(1+\overset{n}{\underset{i=1}{\sum }}x_{i}^{k}\left(t\right)x_{i}^{p}\left(t\right)\right)\text{,}$$

where $L_{1}$ is batch size and $r_{j}^{k}\left(t+1\right)=\{\begin{array}{c} r_{1}^{k},e_{j}^{k}\left(t\right)\geq 0;\\ r_{2}^{k},e_{j}^{k}\left(t\right)<0. \end{array}$

As stated in Golovko et al. (2023), the above expressions Eqs. (1, 2) can significantly increase the learning quality of a single-layer perceptron and achieve an optimal solution to the problem. The proposed approach was generalized to unsupervised pretraining of deep neural network (Golovko et al., 2023), using autoencoder method. The primary goal of the present work is to obtain the analytical expressions to learning rate estimation for restricted Boltzmann machine with ReLU activation function.

## 3 Restricted Boltzmann machine

In this section we consider different representation of RBM from structure and learning point of view.

**Figure 1:**
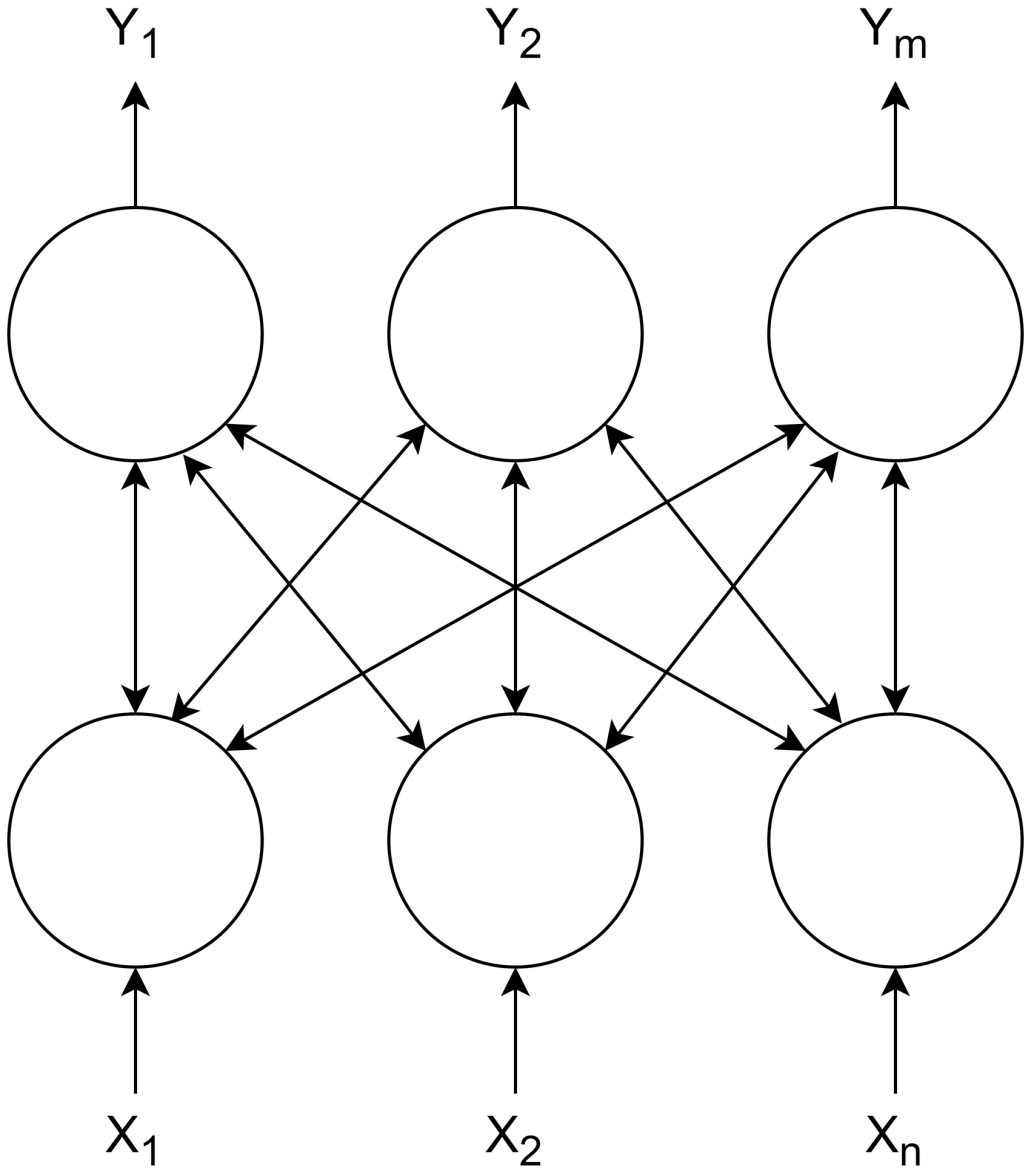
Restricted Boltzmann machine.

Let us consider a conventional restricted Boltzmann machine (Hinton, 2010), which has bipartite structure consisting of two layers: a visible layer containing n units and hidden layer containing m units (Figure 1).

In the RBM structure, each neuron in visible layer is connected to all the units in the hidden layer, using bidirectional weights W. RBM can be used as main building blocks for deep neural networks (Hinton, 2002, 2010; Hinton et al., 2006; Nair and Hinton, 2010). Usually the states of visible and hidden units are defined using a probabilistic version of the sigmoid activation function according to Eqs. (3, 4):


(3) $$p\left(y_{j}|x\right)=\frac{1}{1+e^{-S_{j}}},\;S_{j}=\overset{n}{\underset{i=1}{\sum }}w_{ij}x_{i}+T_{j}$$

(4) $$p\left(x_{i}|x\right)=\frac{1}{1+e^{-S_{i}}},\;S_{i}=\overset{m}{\underset{j=1}{\sum }}w_{ij}y_{j}+T_{i}$$

It should be noted that the variables at the hidden layer are independent given the state of the visible units, and vice versa as shown in expression Eq. (5):


(5) $$P\left(x|y\right)=\overset{n}{\underset{i=1}{\prod }}P\left(x_{i}|y\right)$$

$$P\left(y|x\right)=\overset{m}{\underset{j=1}{\prod }}P\left(y_{j}|x\right)$$

The hidden units of the RBM can be interpreted as feature detectors which capture the regularities of the input data. The traditional way of getting the training rule is to maximize the function of log-likelihood of the input data distribution P(x). In other words, it is necessary to reproduce the distribution of input data as closely as possible using the states of hidden units. The main properties of conventional RBM are the following: symmetric weights in the hidden and visible layers; Gibbs sampling during the training and stochastic neurons. Next, we will consider a RBM that is characterized only by the first two properties, and the neurons are not stochastic.

**Figure 2:**
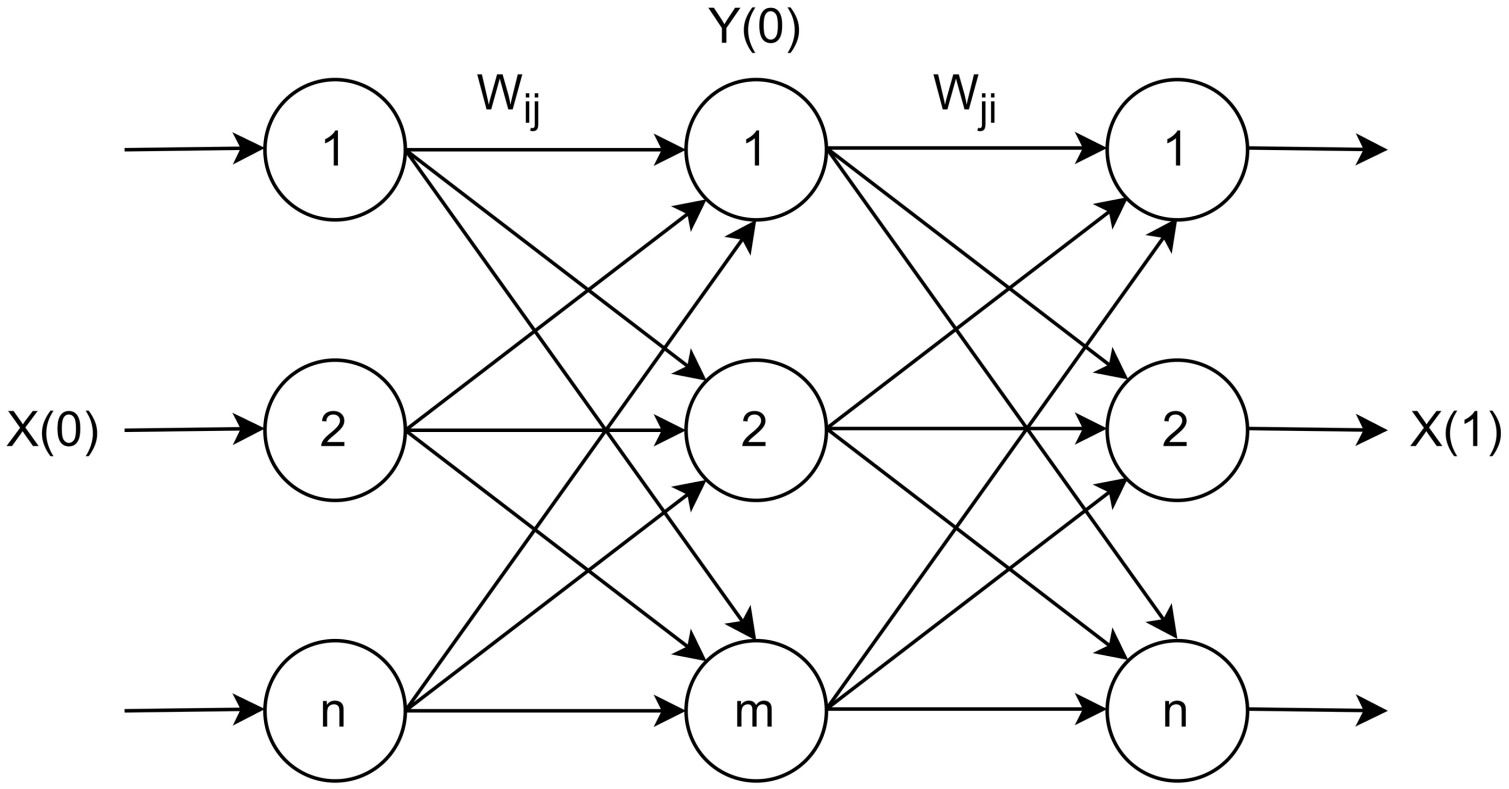
Unfolded representation of RBM.

Let us consider unfolded representation of the RBM using three layers (visible, hidden and visible; Golovko et al., 2015, 2016) as shown in Figure 2. Such a representation of RBM is equivalent to PCA or autoencoder neural network, where the hidden and last visible layer is, respectively, compression and reconstruction layer.

**Figure 3:**
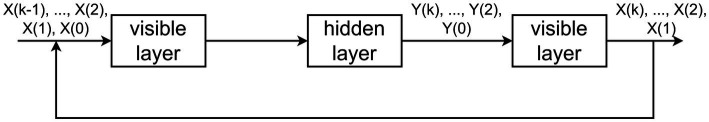
Gibbs sampling.

Let us consider the Gibbs sampling using CD-k. In this case we can represent Gibbs sampling for above structure as shown in Figure 3.

Next, we will consider Gibbs sampling for CD-1. Let x(0) is the input data, that enter at the visible layer at time 0. Then the output of the hidden layer is defined as follows Eqs. (6, 7):


(6) $$y_{j}\left(0\right)=F\left(S_{j}\left(0\right)\right)$$

(7) $$S_{j}\left(0\right)=\underset{i}{\sum }\omega_{ij}x_{i}\left(0\right)+T_{j}$$

The reconstruction layer reproducts the data from the hidden layer. As a result we can obtain x(1) at time 1 using Eqs. (8, 9):


(8) $$x_{i}\left(1\right)=F\left(S_{i}\left(1\right)\right)$$

(9) $$S_{i}\left(1\right)=\underset{j}{\sum }\omega_{ij}y_{j}\left(0\right)+T_{i}$$

After this, x(1) enters the visible layer and we can obtain the output of the hidden layer the following way Eqs. (10, 11):


(10) $$y_{j}\left(1\right)=F\left(S_{j}\left(1\right)\right)$$

(11) $$S_{j}\left(1\right)=\underset{i}{\sum }\omega_{ij}x_{i}\left(1\right)+T_{j}$$

As mentioned before the conventional approach of getting the training rule is to maximize the function of log-likelihood of the input data distribution. In Golovko et al. (2015, 2016), we have proposed an alternative approach in order to obtain RBM learning rule, which is based on the minimization of mean square error (MSE). As stated in Golovko et al. (2016) the primary goal of training RBM is to minimize the reconstruction mean squared error (MSE) in the hidden and visible layers simultaneously. The MSE in the hidden layer is proportional to the difference between the states of the hidden units at the various time steps. Then in case of CD-1 the MSE in the hidden layer is defined as shown in expression Eq. (12):


(12) $$E_{h}\left(1\right)=\frac{1}{2}\overset{L}{\underset{l=1}{\sum }}\overset{m}{\underset{j=1}{\sum }}{\left(y_{j}^{k}\left(1\right)-y_{j}^{k}\left(0\right)\right)}^{2}$$

Similarly, the MSE in the inverse layer is proportional to the difference between the states of the inverse units at the various time steps Eq. (13):


(13) $$E_{v}\left(1\right)=\frac{1}{2}\overset{L}{\underset{l=1}{\sum }}\overset{n}{\underset{i=1}{\sum }}({\left(x_{i}^{k}\left(1\right)-x_{i}^{k}\left(0\right)\right)}^{2}$$

where L is the number of training patterns.

Then the main purpose of the training RBM is to minimize the total mean squared error (MSE), which is defined as the sum of errors Eq. (14):


(14) $$E_{s}=E_{h}\left(1\right)+E_{v}\left(1\right)$$

The following theorem is proved in Golovko et al. (2016).

**Theorem 3:** Maximization of the log-likelihood input data distribution P(x) in the space of synaptic weights of the restricted Boltzmann machine is equivalent to special case of minimizing the reconstruction mean squared error in the same space.

As a result, the following training rule was obtained for online learning Eq. (15):


(15) $$\omega_{ij}\left(t+1\right)=\omega_{ij}\left(t\right)-\alpha \left(\begin{array}{l} \left(y_{j}\left(1\right)-y_{j}\left(0\right)\right)F^{\prime }\left(S_{j}\left(1\right)\right)x_{i}\left(1\right)\\ +\left(x_{i}\left(1\right)-x_{i}\left(0\right)\right)F^{\prime }\left(S_{i}\left(1\right)\right)y_{j}\left(0\right) \end{array}\right)$$

$$T_{i}\left(t+1\right)=T_{i}\left(t\right)-\alpha \left(x_{i}\left(1\right)-x_{i}\left(0\right)\right)F^{\prime }\left(S_{i}\left(1\right)\right)$$

$$T_{j}\left(t+1\right)=T_{j}\left(t\right)-\alpha \left(y_{j}\left(1\right)-y_{j}\left(0\right)\right)F^{\prime }\left(S_{j}\left(1\right)\right)$$

where α is learning rate.

It is easy to show, that if


$$F^{\prime }\left(S_{j}\left(1\right)\right)=\frac{\partial y_{j}\left(1\right)}{\partial S_{j}\left(1\right)}=F^{\prime }(S_{i}\left(1\right)=\frac{\partial x_{i}\left(1\right)}{\partial S_{i}\left(1\right)}=1\text{,}$$

then can be obtained the conventional learning rule Eq. (16):


$$\omega_{ij}\left(t+1\right)=\omega_{ij}\left(t\right)+\alpha \left(x_{i}\left(0\right)y_{j}\left(0\right)-x_{i}\left(1\right)y_{j}\left(1\right)\right)\text{,}$$

(16) $$T_{j}\left(t+1\right)=T_{j}\left(t\right)+\alpha \left(y_{j}\left(0\right)-y_{j}\left(1\right)\right)$$

$$T_{i}\left(t+1\right)=T_{i}\left(t\right)+\alpha \left(x_{i}\left(0\right)-x_{i}\left(1\right)\right)\text{.}$$

We have seen in this section, that depending on the loss function can be obtained different learning rules with derivatives and without derivatives of activation function with respect to weighted sum. In further we will use the learning rule with derivatives.

## 4 Learning of RBM with ReLU

In this section, we consider the definition of ReLU activation function and RBM learning rule. As noted earlier, we consider RBM with deterministic neurons and for learning we will use the expressions given in the previous section. First of all, let us define the ReLU activation function by the following way.

Definition: The ReLU activation function for j-th unit can be presented by the following way Eq. (17):


(17) $$y_{j}\left(t\right)=F\left(S_{j}\left(t\right)\right)=r_{j}\left(t\right)S_{j}\left(t\right)$$

where $r_{j}\left(t\right)$ is defined by the following way Eq. (18):


(18) $$r_{j}\left(t\right)=\{\begin{array}{c} r_{1},S_{j}\left(t\right)\geq 0;\\ r_{2},S_{j}\left(t\right)<0. \end{array}$$

Here $r_{1}$ and $r_{2}$ denote corresponding slopes of the ReLU function; $r_{1}\neq r_{2}$ ; $0\leq r_{2}<1.$ Usually $r_{1}=1$ is used.

The above definition of the activation function allows the use of any slope of straight lines and generalizes the conventional definition of ReLU and leaky ReLU activation functions.

Then we can obtain the following derivatives Eq. (19):


(19) $$\begin{array}{l} F^{\prime }\left(S_{j}\left(1\right)\right)=\frac{\partial y_{j}\left(1\right)}{\partial S_{j}\left(1\right)}=r_{j}\left(1\right)\;\mathrm{and}\\ \;F^{\prime }\left(S_{i}\left(1\right)\right)=\frac{\partial x_{i}\left(1\right)}{\partial S_{i}\left(1\right)}=r_{i}\left(1\right) \end{array}$$

Using the previous results Eq. (15), we can write the following equations for online learning Eq. (20):


(20) $$\omega_{ij}\left(t+1\right)=\omega_{ij}\left(t\right)-\alpha \left(\begin{array}{l} \left(y_{j}\left(1\right)-y_{j}\left(0\right)\right)r_{j}\left(1\right)x_{i}\left(1\right)\\ +\left(x_{i}\left(1\right)-x_{i}\left(0\right)\right)r_{i}\left(1\right)y_{j}\left(0\right) \end{array}\right)$$

$$T_{i}\left(t+1\right)=T_{i}\left(t\right)-\alpha \left(x_{i}\left(1\right)-x_{i}\left(0\right)\right)r_{i}\left(1\right)$$

$$T_{j}\left(t+1\right)=T_{j}\left(t\right)-\alpha \left(y_{j}\left(1\right)-y_{j}\left(0\right)\right)r_{j}\left(1\right)$$

If we apply the batch learning and batch size is $L_{1}$ ,


(21) $$\omega_{ij}\left(t+1\right)=\omega_{ij}\left(t\right)-\alpha \overset{L_{1}}{\underset{p=1}{\sum }}\left(\begin{array}{l} \left(y_{j}^{p}\left(1\right)-y_{j}^{p}\left(0\right)\right)r_{j}^{p}\left(1\right)x_{i}^{p}\left(1\right)\\ +\left(x_{i}^{p}\left(1\right)-x_{i}^{p}\left(0\right)\right)r_{i}^{p}\left(1\right)y_{j}^{p}\left(0\right) \end{array}\right)$$

$$T_{i}\left(t+1\right)=T_{i}\left(t\right)-\alpha \overset{L_{1}}{\underset{p=1}{\sum }}\left(x_{i}^{p}\left(1\right)-x_{i}^{p}\left(0\right)\right)r_{i}^{p}\left(1\right)$$

$$T_{j}\left(t+1\right)=T_{j}\left(t\right)-\alpha \overset{L_{1}}{\underset{p=1}{\sum }}\left(y_{j}^{p}\left(1\right)-y_{j}^{p}\left(0\right)\right)r_{j}^{p}\left(1\right)$$

Thus, in this section, we have derived learning rules for RBM with ReLU activation function. Further we will use given above expressions Eq. (21) for RBM learning.

## 5 Materials and methods

In this section we address adaptive learning rate estimation for RBM with ReLU activation function. Since the RBM network has symmetric weights in the hidden and visible layers, we should derive the optimal training step for the two layers.

The learning step is called adaptive, which is chosen at each stage of the algorithm in such a way in order to minimize the total mean squared error (Golovko et al., 2000; Golovko, 2003; Golovko et al., 2023). We will use the steepest descent approach in order to obtain the expression for adaptive learning rate. Accordingly, to steepest descent approach, the learning step α is selected so as to minimize the mean square error of the new parameters Eq. (22):


(22) $$\alpha \left(t\right)=\min_{¯}E_{s}\left(y_{j}^{k}\left(1,t+1\right),x_{i}^{k}\left(1,t+1\right)\right)$$

where $y_{j}^{k}\left(1,t+1\right),x_{i}^{k}\left(1,t+1\right)$ are the outputs of the hidden and visible layer at the next time t+1 after updating the RBM trainable parameters.

As a result, at each step of learning algorithm we should choose the value of learning rate in such a way that, when modifying weights and thresholds to guarantee a minimum of the mean squared error for each batch or each example from the training data set.

**Theorem 4:** For an RBM network with a ReLU activation function in the case of batch learning, the value of the adaptive learning step, that minimizes the mean squared error for each batch is calculated based on the following expression:


(23) $$\alpha \left(t\right)=\frac{\sum_{k=1}^{L_{1}}\sum_{j=1}^{m}c_{j}^{k}+\sum_{k=1}^{L_{1}}\sum_{i=1}^{n}c_{i}^{k}}{\begin{array}{l} \sum_{k=1}^{L_{1}}\sum_{j=1}^{m}{\left(r_{j}^{k}\left(1,t+1\right)\right)}^{2}{\left(b_{j}^{k}\right)}^{2}\\ +\sum_{k=1}^{L_{1}}\sum_{i=1}^{n}{\left(r_{i}^{k}\left(1,t+1\right)\right)}^{2}{\left(b_{i}^{k}\right)}^{2} \end{array}}$$

where the corresponding terms are determined according to the expressions Eqs. (24–32):


(24) $$c_{j}^{k}=\left(r_{j}^{k}\left(1,t+1\right)S_{j}^{k}\left(1\right)-y_{j}^{k}\left(0\right)\right)r_{j}^{k}\left(1,t+1\right)b_{j}^{k}$$

(25) $$c_{i}^{k}=\left(r_{i}^{k}\left(1,t+1\right)S_{i}^{k}\left(1\right)-x_{i}^{k}\left(0\right)\right)r_{i}^{k}\left(1,t+1\right)b_{i}^{k}$$

(26) $$b_{j}^{k}=f_{j}^{k}+z_{j}^{k}$$

(27) $$f_{j}^{k}=\overset{L_{1}}{\underset{p=1}{\sum }}r_{j}^{p}\left(1\right)\left(y_{j}^{p}\left(1\right)-y_{j}^{p}\left(0\right)\right)\left(1+\overset{n}{\underset{i=1}{\sum }}x_{i}^{k}\left(1\right)x_{i}^{p}\left(1\right)\right)$$

(28) $$z_{j}^{k}=\overset{L_{1}}{\underset{p=1}{\sum }}y_{j}^{p}\left(0\right)\overset{n}{\underset{i=1}{\sum }}x_{i}^{k}\left(1\right)\left(x_{i}^{p}\left(1\right)-x_{i}^{p}\left(0\right)\right)r_{i}^{p}\left(1\right)$$

(29) $$b_{i}^{k}=f_{i}^{k}+z_{i}^{k}\text{,}$$

(30) $$f_{i}^{k}=\overset{L_{1}}{\underset{p=1}{\sum }}r_{i}^{p}\left(1\right)\left(x_{i}^{p}\left(1\right)-x_{i}^{p}\left(0\right)\right)\left(1+\overset{m}{\underset{j=1}{\sum }}y_{j}^{k}\left(0\right)y_{j}^{p}\left(0\right)\right)$$

(31) $$z_{i}^{k}=\overset{L_{1}}{\underset{p=1}{\sum }}x_{i}^{p}\left(1\right)\overset{m}{\underset{j=1}{\sum }}y_{j}^{k}\left(0\right)\left(y_{j}^{p}\left(1\right)-y_{j}^{p}\left(0\right)\right)r_{j}^{p}\left(1\right)$$

(32) $$r_{j}^{k}\left(1,t+1\right)=\{\begin{array}{c} r_{1},y_{j}^{k}\left(0\right)\geq 0;\\ r_{2},y_{j}^{k}\left(0\right)<0, \end{array}\;r_{i}^{k}\left(1,t+1\right)=\{\begin{array}{c} r_{1},x_{i}^{k}\left(0\right)\geq 0;\\ r_{2},x_{i}^{k}\left(0\right)<0. \end{array}$$

**Proof:** We should find adaptive learning rate by minimizing the following loss function Eq. (33):


(33) $$\begin{array}{c} E_{s}=\frac{1}{2}\overset{L_{1}}{\underset{k=1}{\sum }}\overset{m}{\underset{j=1}{\sum }}{\left(y_{j}^{k}\left(1,t+1\right)-y_{j}^{k}\left(0\right)\right)}^{2}\\ \;+\frac{1}{2}\overset{L_{1}}{\underset{k=1}{\sum }}\overset{n}{\underset{i=1}{\sum }}{\left(x_{i}^{k}\left(1,t+1\right)-x_{i}^{k}\left(0\right)\right)}^{2} \end{array}$$

The output of the hidden and visible layer at the next time t+1 after updating trainable parameters can be defined as according to the expressions Eq. (34):


(34) $$\begin{array}{l} x_{i}^{k}\left(1,t+1\right)=r_{i}^{k}\left(1,t+1\right)S_{i}^{k}\left(1,t+1\right),\\ y_{j}^{k}\left(1,t+1\right)=r_{j}^{k}\left(1,t+1\right)S_{j}^{k}\left(1,t+1\right) \end{array}$$

Let us consider at the beginning the weighted sum of the hidden layer at the next time t+1


(35) $$S_{j}^{k}\left(1,t+1\right)=\overset{n}{\underset{i=1}{\sum }}\omega_{ij}\left(t+1\right)x_{i}^{k}\left(1\right)+T_{j}\left(t+1\right)$$

Substituting corresponding expression for weights and threshold updating from Eq. (21) in Eq. (35) we can obtain Eq. (36)


(36) $$S_{j}^{k}\left(1,t+1\right)=S_{j}^{k}\left(1\right)-\alpha b_{j}^{k}$$

where *bj* is defined using Eq. (37)


(37) $$\begin{array}{c} b_{j}^{k}=\overset{L_{1}}{\underset{p=1}{\sum }}r_{j}^{p}\left(1\right)\left(y_{j}^{p}\left(1\right)-y_{j}^{p}\left(0\right)\right)\left(1+\overset{n}{\underset{i=1}{\sum }}x_{i}^{k}\left(1\right)x_{i}^{p}\left(1\right)\right)\\ \;+\overset{L_{1}}{\underset{p=1}{\sum }}y_{j}^{p}\left(0\right)\overset{n}{\underset{i=1}{\sum }}x_{i}^{k}\left(1\right)\left(x_{i}^{p}\left(1\right)-x_{i}^{p}\left(0\right)\right)r_{i}^{p}\left(1\right) \end{array}$$

We will use a similar approach for the visible layer. Then the weighted sum of the visible layer can be defined as follows:


(38) $$S_{i}^{k}\left(1,t+1\right)=\overset{m}{\underset{j=1}{\sum }}\omega_{ij}\left(t+1\right)y_{j}^{k}\left(0\right)+T_{i}\left(t+1\right)$$

Substituting corresponding expression from Eq. (21) in Eq. (38) we can write obtain Eq. (39)


(39) $$S_{i}^{k}\left(1,t+1\right)=S_{i}^{k}\left(1\right)-\alpha b_{i}^{k}$$

Where *bj* is defined using Eq. (40)


(40) $$\begin{array}{c} b_{i}^{k}=\overset{L_{1}}{\underset{p=1}{\sum }}r_{i}^{p}\left(1\right)\left(x_{i}^{p}\left(1\right)-x_{i}^{p}\left(0\right)\right)\left(1+\overset{m}{\underset{j=1}{\sum }}y_{j}^{k}\left(0\right)y_{i}^{p}\left(0\right)\right)\\ \;+\overset{L_{1}}{\underset{p=1}{\sum }}x_{i}^{p}\left(1\right)\overset{m}{\underset{j=1}{\sum }}y_{j}^{k}\left(0\right)\left(y_{j}^{p}\left(1\right)-y_{j}^{p}\left(0\right)\right)r_{j}^{p}\left(1\right) \end{array}$$

As a result, we can obtain the final expressions regarding output of the hidden and visible layer Eq. (41)


(41) $$y_{j}^{k}\left(1,t+1\right)=r_{j}^{k}\left(1,t+1\right)\left(S_{j}^{k}\left(1\right)-\alpha b_{j}^{k}\right)$$

$$x_{i}^{k}\left(1,t+1\right)=r_{i}^{k}\left(1,t+1\right)\left(S_{i}^{k}\left(1\right)-\alpha b_{i}^{k}\right)\text{.}$$

Differentiating the loss function $E_{s}$ with respect to α we can obtain Eq. (42)


(42) $$\begin{array}{l} \frac{dE_{s}}{d\alpha }=\overset{L_{1}}{\underset{k=1}{\sum }}\overset{m}{\underset{j=1}{\sum }}\left(r_{j}^{k}\left(1,t+1\right)S_{j}^{k}\left(1\right)-\alpha r_{j}^{k}\left(1,t+1\right)b_{j}^{k}-y_{j}^{k}\left(0\right)\right)\\ \;\left(-r_{j}\left(1,t+1\right)b_{j}\right)+\overset{L_{1}}{\underset{k=1}{\sum }}\overset{n}{\underset{i=1}{\sum }}\left(\begin{array}{l} r_{i}^{k}\left(t+1\right)S_{i}^{k}\left(1\right)\\ -\alpha r_{i}^{k}\left(1,t+1\right)b_{i}^{k}-x_{i}^{k}\left(0\right) \end{array}\right)\\ \;\left(-r_{i}^{k}\left(1,t+1\right)b_{i}^{k}\right)=0\; \end{array}$$

As a result, we can obtain the following final expression Eq. (43):


(43) $$\alpha \left(t\right)=\frac{\begin{array}{l} \sum_{k=1}^{L_{1}}\sum_{j=1}^{m}\left(r_{j}^{k}\left(1,t+1\right)S_{j}^{k}\left(1\right)-y_{j}^{k}\left(0\right)\right)r_{j}^{k}\left(1,t+1\right)b_{j}^{k}\\ +\sum_{k=1}^{L_{1}}\sum_{i=1}^{n}\left(r_{i}^{k}\left(1,t+1\right)S_{i}^{k}\left(1\right)-x_{i}^{k}\left(0\right)\right)r_{i}^{k}\left(1,t+1\right)b_{i}^{k} \end{array}}{\begin{array}{l} \sum_{k=1}^{L_{1}}\sum_{j=1}^{m}{\left(r_{j}^{k}\left(1,t+1\right)\right)}^{2}{\left(b_{j}^{k}\right)}^{2}\\ +\sum_{k=1}^{L_{1}}\sum_{i=1}^{n}{\left(r_{i}^{k}\left(1,t+1\right)\right)}^{2}{\left(b_{i}^{k}\right)}^{2} \end{array}}$$

Since in accordance with Eq. (44)


(44) $$\frac{d^{2}E_{s}}{d^{2}\alpha }>0$$

we have found the minimum of the cost function. Thus the theorem is proved.

As follows from the proven theorem, the adaptive learning rate minimizes the mean squared error of the network under updating weights and thresholds.

The major difficulty arises in the computing of $r_{j}^{k}\left(1,t+1\right),r_{i}^{k}\left(1,t+1\right)$ , because it is desired parameters of ReLU transfer function. Since the desired outputs of the hidden and visible layer correspondingly $y_{j}^{k}\left(0\right)$ and $x_{i}^{k}\left(0\right)$ then we can write Eq. (45)


(45) $$r_{j}^{k}\left(1,t+1\right)=\{\begin{array}{c} r_{1},y_{j}^{k}\left(0\right)\geq 0;\\ r_{2},y_{j}^{k}\left(0\right)<0, \end{array}\;r_{i}^{k}\left(1,t+1\right)=\{\begin{array}{c} r_{1},x_{i}^{k}\left(0\right)\geq 0;\\ r_{2},x_{i}^{k}\left(0\right)<0. \end{array}$$

**Theorem 5:** For RBM network with a ReLU activation function in the case of online learning, the value of the adaptive learning step, that minimizes the mean squared error for each pattern is defined as follows Eq. (46):


(46) $$\alpha \left(t\right)=\frac{\sum_{j=1}^{m}c_{j}+\sum_{i=1}^{n}c_{i}}{\sum_{j=1}^{m}r_{j}^{2}\left(1,t+1\right)b_{j}^{2}+\sum_{i=1}^{n}r_{i}^{2}\left(1,t+1\right)b_{i}^{2}}$$

where the corresponding terms are calculated based on Eqs. (47–55)


(47) $$c_{j}=\left(r_{j}\left(1,t+1\right)S_{j}\left(1\right)-y_{j}\left(0\right)\right)r_{j}\left(1,t+1\right)b_{j}$$

(48) $$c_{i}=\left(r_{i}\left(1,t+1\right)S_{i}\left(1\right)-x_{i}\left(0\right)\right)r_{i}\left(1,t+1\right)b_{i}$$

(49) $$b_{j}=f_{j}+z_{j}$$

(50) $$f_{j}=r_{j}\left(1\right)\left(y_{j}\left(1\right)-y_{j}\left(0\right)\right)\left(1+\overset{n}{\underset{i=1}{\sum }}x_{i}^{2}\left(1\right)\right)$$

(51) $$z_{j}=y_{j}\left(0\right)\overset{n}{\underset{i=1}{\sum }}x_{i}\left(1\right)\left(x_{i}\left(1\right)-x_{i}\left(0\right)\right)r_{i}\left(1\right)$$

(52) $$b_{i}=f_{i}+z_{i}$$

(53) $$f_{i}=r_{i}\left(1\right)\left(x_{i}\left(1\right)-x_{i}\left(0\right)\right)\left(1+\overset{m}{\underset{j=1}{\sum }}y_{j}^{2}\left(0\right)\right)$$

(54) $$z_{i}=x_{i}\left(1\right)\overset{m}{\underset{j=1}{\sum }}y_{j}\left(0\right)\left(y_{j}\left(1\right)-y_{j}\left(0\right)\right)r_{j}\left(1\right)$$

(55) $$r_{j}\left(1,t+1\right)=\{\begin{array}{c} r_{1},y_{j}\left(0\right)\geq 0;\\ r_{2},y_{j}\left(0\right)<0, \end{array}\;r_{i}\left(1,t+1\right)=\{\begin{array}{c} r_{1},x_{i}\left(0\right)\geq 0;\\ r_{2},x_{i}\left(0\right)<0. \end{array}$$

This theorem is proved by the same approach.

It should be noted that the proposed expressions for calculating the learning step are valid when $r_{2}\neq 0.$ If $r_{2}=0$ , then in accordance with RBM learning rule Eq. (21), the training is performed only in the area where weighted sum is greater than 0, since the gradient of this function is 0, if weighted sum less than 0. In that case, we can simplify the expressions for learning rate.

**Corollary:** For RBM network with a ReLU activation function and $r_{1}=1,r_{2}=0$ in the case of online learning, the value of the adaptive learning step is calculated based on the following expression Eqs. (56–58):


(56) $$\alpha \left(t\right)=\frac{\sum_{j=1}^{m}\left(y_{j}\left(1\right)-y_{j}\left(0\right)\right)b_{j}+\sum_{i=1}^{n}\left(x_{i}\left(1\right)-x_{i}\left(0\right)\right)b_{i}}{\sum_{j=1}^{m}b_{j}^{2}+\sum_{i=1}^{n}b_{i}^{2}}$$

Where


(57) $$b_{j}=\left(y_{j}\left(1\right)-y_{j}\left(0\right)\right)-\overset{n}{\underset{i=1}{\sum }}x_{i}\left(1\right)(\left(y_{j}\left(0\right)x_{i}\left(0\right)-x_{i}\left(1\right)y_{j}\left(1\right)\right)$$

(58) $$b_{i}=\left(x_{i}\left(1\right)-x_{i}\left(1\right)\right)-\overset{m}{\underset{j=1}{\sum }}y_{j}\left(0\right)(\left(y_{j}\left(0\right)x_{i}\left(0\right)-x_{i}\left(1\right)y_{j}\left(1\right)\right)$$

In a similar way, we can obtain an expression for the adaptive step calculation when using batch learning. The proposed expressions allow to estimate the learning rate after presenting every batch or pattern to the neural network and based on the minimization of loss function. Adaptive training step approach permits to choose automatically step for every batch or pattern from training data set. The performance of proposed approach is discussed in the next section.

## 6 Experiments

This section summarizes numerical results obtained by the application of adaptive and constant learning rate. In order to evaluate the performance of the proposed approach we will conduct various experiments using RBM and deep neural network. In all experiments, we will use batch learning with adaptive rate Eq. (23). In that case the weights and thresholds of the network will be modified based on rule Eq. (21) presented in this paper. For experiments, we will use both an artificial and the MNIST dataset. The primary aim of this section is to compare learning of neural network with and without proposed training approach with adaptive learning rate. The experiments are divided into 2 groups. The first experiments focuses on the RBM network and the second on deep multilayer neural network.

### 6.1 RBM results

Let us consider the use of an adaptive learning step for a RBM network. To evaluate the effectiveness of adaptive learning rate we will use two datasets.

#### 6.1.1 Artificial dataset

The artificial data x lie on a one-dimensional manifold (a helical loop) embedded in three dimensions (Scholz et al., 2008) and were generated from a uniformly distributed factor t in the range [0.05, 0.95]:


$$\left\{ \begin{array}{l} x_{1}=\sin\left(\pi t\right)+\mu \;,\\ x_{2}=\cos\left(\pi t\right)+\mu \\ x_{3}=t+\mu \;. \end{array}\;\text{,}\right.$$

where μ – Gaussian noise with mean 0 and standard deviation 0.05.

**Figure 4:**
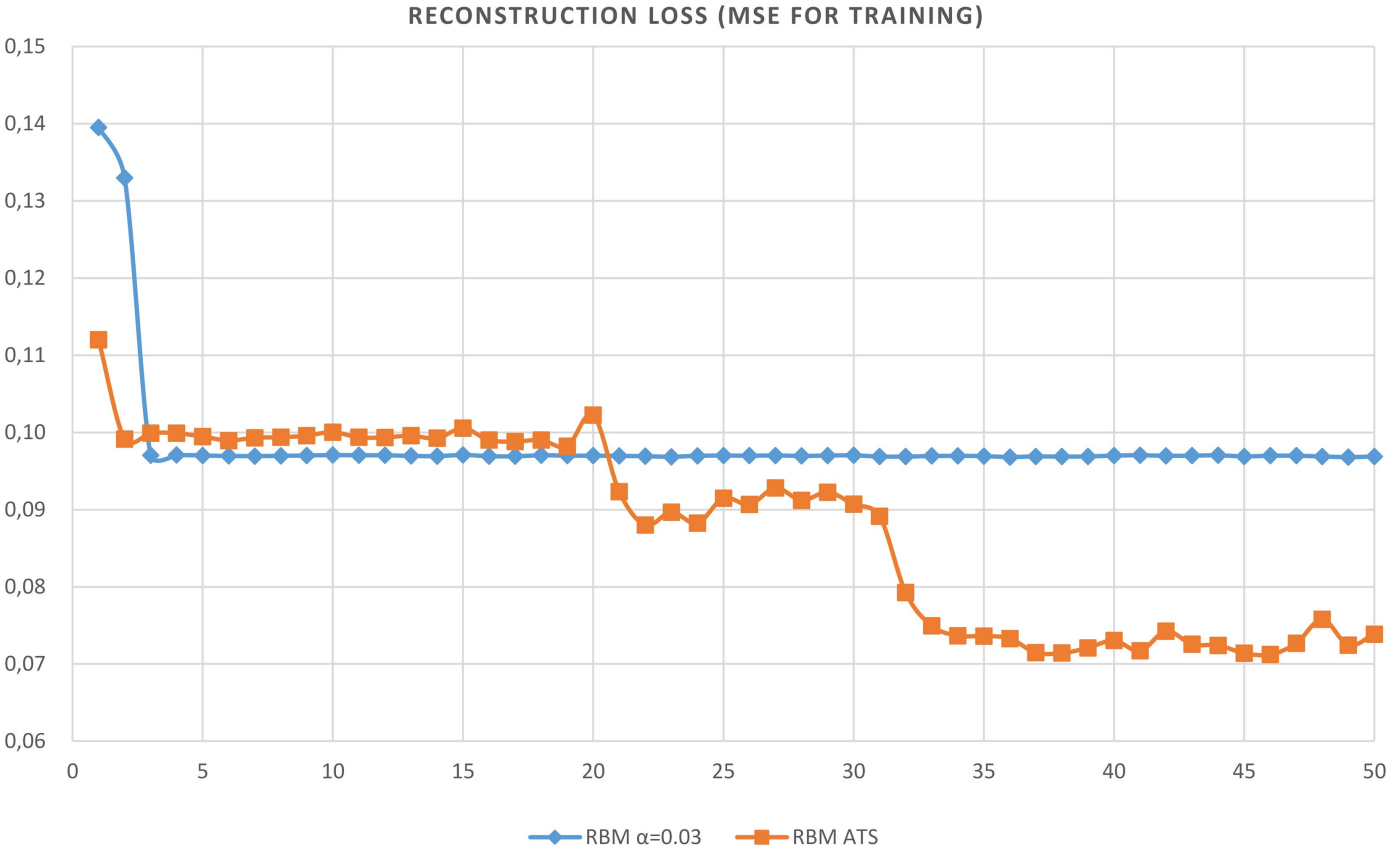
Plot of reconstruction accuracy versus epoch for learning using adaptive and constant learning rates.

**Figure 5:**
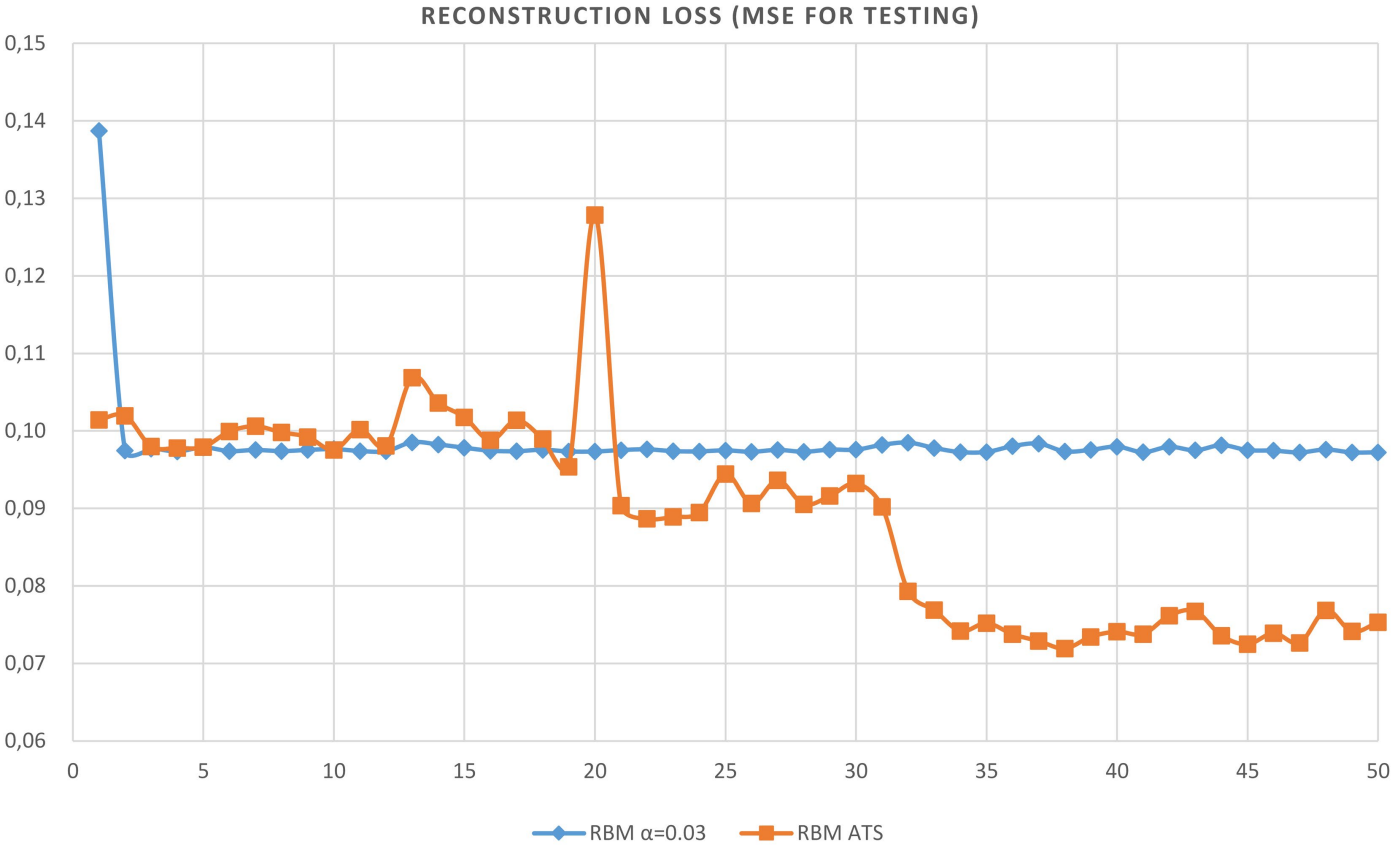
Plot of reconstruction accuracy versus epoch for testing using adaptive and constant learning rates.

The primary goal of the experiment is to study the performance of ATS for data compression and reconstruction. Then the RBM will consist of 3 visible and 1 hidden unit. The training dataset consists of 1,000 samples. The size of the test patterns is also 1,000. The batch size equal 8 and the parameters of ReLU function are the following: $r_{1}=1,r_{2}=0.01\text{.}$ We trained the RBM network using only clean data and tested using noisy data. The evolution of reconstruction error vs. epoch of RBM learning is provided in Table 1. As can be seen from the table the adaptive learning rate has obvious excellence compared to constant steps. The plots of the reconstruction accuracy vs. epoch for learning and testing using the best constant and adaptive rate are presented in Figures 4, 5. It should be noted here that testing is performed after each learning epoch. As follows from the presented figures, the adaptive learning rate has the evident advantage compared to the fixed learning rate, namely, the best performance in terms of learning quality and generalization ability.

**Table 1:** Evolution of the reconstruction error (MSE) for artificial data.

Number of epochs	α = 3e − 1	α = 3e − 2	α = 3e − 3	α = 3e − 4	α = 3e − 5	ATS
10	0.2223	0.0974	0.1394	0.1393	0.1862	0.1001
20	0.2240	0.0975	0.0982	0.1392	0.1653	0.0903
30	0.2241	0.0982	0.0983	0.1392	0.1537	0.0902
40	0.2230	0.0972	0.0982	0.1392	0.1474	0.0737
50	0.2231	0.0972	0.0982	0.1391	0.1422	0.0753

#### 6.1.2 MNIST dataset

In this section we will use the MNIST dataset, which contains 60,000 hand-written digit images for training, and 10,000 images for testing. Data in MNIST are grayscale images with size 28 × 28. Before training the images are normalized to be zero-mean.

Let us model the RBM network. This simulation is used to illustrate the compression and reconstruction properties of restricted Boltzmann machine. Let us model the RBM network which consist of 784 neurons of visible and 128 units of hidden layers. The main goal of such modeling is to compress and reconstruct MNIST data. The parameters of experiments are shown in Table 2. We used original images from MNIST dataset and before representation to RBM only centering is performed.

**Table 2:** Parameters of experiments.

Number of pretraining epoch	Batch size	$r_{1}$	$r_{2}$
8	128	1	0.01

The evolution of reconstruction square error Eq. (13) is shown in Table 3. Here div. Denotes divergence of learning. The analysis of the data in this table indicates that learning with a constant rate is unstable. For instance, if α = 1e − 4, the neural network cannot be trained. As can be seen only training with a constant learning rate (3e − 7) leads to a positive result.

**Table 3:** Evolution of the reconstruction error (MSE) for MNIST.

Number of epochs	α = 1e − 4	α = 3e − 5	α = 3e − 6	α = 3e − 7	ATS
1	0,126	0.095	0.073	0.073787208	0.070819163
2	0,178	0.111	0.074	0.073663836	0.070752306
3	0,213	0.125	0.075	0.073577446	0.070733909
4	div.	0.138	0.077	0.073518835	0.070713023
5	div.	0.154	0.081	0.073481718	0.070700173
6	div.	0.171	0.087	0.073461681	0.070700861
7	div.	0.199	0.098	0.073455503	0.070687373
8	div.	0.232	0.115	0.073460822	0.070678658

Hence, the learning algorithms with constant training step can diverge if the learning parameters are not chosen appropriately, as shown in Table 3. Therefore, we should select the constant learning rate very carefully.

**Figure 6:**
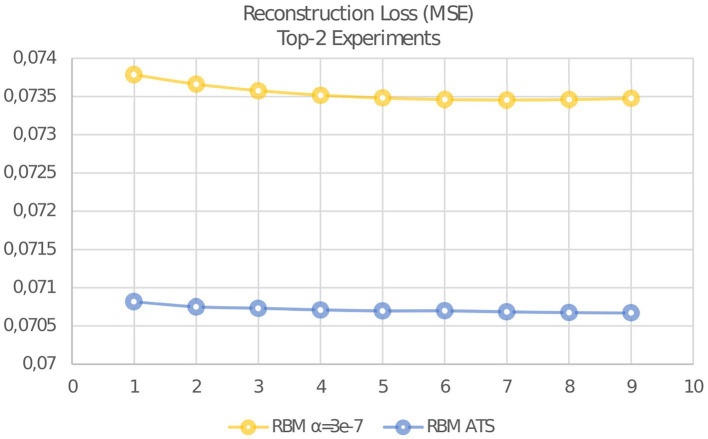
Evolution of reconstruction error MSE for adaptive and constant learning rates.

Also it should be remarked, that learning with ATS have shown the result after first epoch better than with constant step at any epoch. After 8 epochs have obtained the best result with reconstruction error of 0.070678658. The best result using constant step is 0.073455503. This result was obtained after 8 epochs. As can be seen, the adaptive learning rate has a significant advantage in comparison with the constant learning stage. The evolution of reconstruction error is presented in Figure 6.

### 6.2 Deep multilayer perceptron

Let us consider the analysis of the proposed approach for a deep multilayer neural network using the MNIST dataset. We have used for experiments deep perceptron with ReLU activation function which has the following structure: 784-1600-1600-800-800-10. The parameters of experiments are shown in Table 4. The results of our experiments are shown in Table 5. Pretraining is performed using only 1–3 epochs.

**Table 4:** MNIST Classification Experiment Parameters.

Number of pretraining epochs	Number of fine-tuning epochs	Batch size	$r_{1}$	$r_{2}$	Initial α at the finetuning stage
3	50	128	1	0.01	3e − 4

**Table 5:** Testing a deep multilayer perceptron pertaining.

	α = 1e − 4	α = 3e − 5	α = 3e − 6	α = 3e − 7	ADAM	ATS
MSE	div	div	0.0024235	0.0024619	0.0024729	0.0022999
Precision macro	0.0098	0.0098	0.986	0.98562	0.98539	0.98656
Accuracy	0.0098	0.0098	0.986	0.9857	0.9854	0.9866

**Figure 7:**
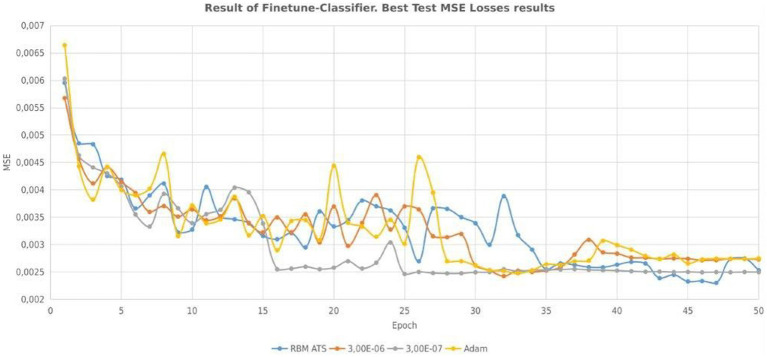
Evolution of MSE for adaptive and constant learning rates.

The evolution of mean squared error for different approaches is presented in Figure 7. Finally, we have the following experimental results, which are shown in Table 5. The smallest test error without using ATS and pretraining is 0.002531. If we use ATS the smallest test error is 0.002299. The smallest test error for pretraining with constant step is 0.002392. As in the previous case, the table shows that learning with a constant rate can be unstable. As a result, a learning algorithm with a constant learning step may be divergent. Tables 6–8 show the predictive performance of different learning approaches for MNIST classification. As can be seen, in general the ATS approach outperformed the learning technique with fixed learning rate. So, for instance, the number of correct predictions using the adaptive rate (Table 6) is 6 and 9 more, respectively, compared to models with a constant step (Tables 7, 8). As can be seen the use of ATS permits to reduce the test error and correspondingly improve the generalization ability.

**Table 6:** Confusion matrix for MNIST classification using pretraining with ATS.

		Predicted values									
		0	1	2	3	4	5	6	7	8	9
Actual values	0	974	0	0	0	0	0	1	2	2	1
	1	0	1,130	0	1	0	1	1	2	0	0
	2	2	0	1,015	2	2	0	0	5	5	1
	3	0	0	1	998	0	3	0	4	3	1
	4	1	0	1	1	966	0	2	1	0	10
	5	2	0	0	5	0	877	2	1	4	1
	6	4	2	0	0	1	4	947	0	0	0
	7	1	2	6	0	0	0	0	1,013	2	4
	8	1	0	1	3	1	0	0	5	960	3
	9	1	2	1	4	7	3	0	4	1	986

**Table 7:** Confusion matrix for MNIST classification using pretraining with α = 3e − 6.

		Predicted values									
		0	1	2	3	4	5	6	7	8	9
Actual values	0	973	1	1	0	0	0	3	1	1	0
	1	0	1,126	1	2	0	2	2	1	1	0
	2	1	1	1,018	2	1	0	1	7	1	0
	3	0	0	3	999	0	2	0	2	2	2
	4	1	2	1	0	969	0	2	0	0	7
	5	2	0	0	5	1	878	2	1	1	2
	6	5	2	0	0	3	2	945	0	1	0
	7	1	2	7	1	1	0	0	1,010	3	3
	8	4	0	3	7	2	1	1	4	950	2
	9	3	2	0	0	6	3	0	3	0	992

**Table 8:** Confusion matrix for MNIST classification using pretraining with α = 3e-7.

		Predicted values									
		0	1	2	3	4	5	6	7	8	9
Actual values	0	974	1	0	1	0	1	1	1	1	0
	1	0	1,126	3	0	0	2	3	0	1	0
	2	0	1	1,021	2	1	0	1	4	2	0
	3	0	0	4	999	0	1	0	1	0	5
	4	1	1	3	0	964	0	3	3	0	7
	5	2	0	0	3	1	881	1	1	2	1
	6	6	2	0	1	4	4	940	0	1	0
	7	2	1	5	3	0	0	0	1,015	2	0
	8	3	0	2	6	1	3	1	2	952	4
	9	4	2	0	3	6	4	0	3	2	985

## 7 Conclusion and discussion

The learning of neural networks is a tricky task, which highly depends on suitable hyperparameters selection to achieve significant performance of a neural network. The choice of an appropriate learning rate is of great importance because it has a significant impact on the training efficiency. Depending on this parameter the learning process can be divergent or convergent.

We have not found any works as concerns exact analytical expressions for the learning rate estimation, based on the steepest descent technique. Such precise analytical expressions can only be obtained for linear and ReLU activation functions. When using the sigmoid activation function, we can only receive approximate expressions for the learning rate using the Taylor series expansion. Since this is a very complicated problem, most of the scientists use the steepest descent method together with the line search approach.

Our previous work (Golovko et al., 2023) reported an adaptive learning rate for a single-layer perceptron with a ReLU activation function. In this work, we extended this idea to obtain the learning rate for the RBM network. As a result, novel analytical expressions for learning step estimation have been proposed in this paper. The proposed approach for ATS estimation is based on minimization the mean squared error for each batch or each sample. The presented expressions are applied for restricted Boltzmann machine learning with ReLU activation function. We consider quasi-conventional RBM, namely we use symmetric weights in the hidden and visible layers, Gibbs sampling and deterministic units. We first demonstrate the proposed approach for ATS calculation is more effective and more efficient for RBM learning than the conventional RBM algorithm. Second, we show that such kind of RBM can be used for deep neural network pretraining using greedy layer wise algorithm. As a result, we can reach better generalization ability.

The main advantages of the proposed approach are the following: it is based on precise mathematical expressions obtained by minimizing the mean squared error for each batch or each pattern; it is capable of automatically defining and adjusting the learning rate during neural network training; it guarantees convergence to well-performing local minima. The disadvantage of the presented approach is higher computational complexity compared to constant step.

This work opens the way toward the following future research: define the conditions where such an learning rate can guarantee convergence to best-performing local minima and to study how this approach can be extended to train a multilayer neural network without pretraining using RBM.

## Data availability statement

Publicly available datasets were analyzed in this study. This data can be found at: https://yann.lecun.com/exdb/mnist/.

## Author contributions

CC: Data curation, Funding acquisition, Investigation, Writing – original draft. VG: Conceptualization, Formal analysis, Supervision, Writing – original draft. AK: Methodology, Project administration, Visualization, Writing – review & editing. EM: Software, Validation, Writing – review & editing. MC: Funding acquisition, Investigation, Writing – review & editing. PL: Methodology, Resources, Software, Writing – review & editing.
